# Correction: Martínez-Murcia et al. SARS-CoV-2 Variants Identification; A Fast and Affordable Strategy Based on Partial S-Gene Targeted PCR Sequencing. *Viruses* 2022, *14*, 2588

**DOI:** 10.3390/v15030771

**Published:** 2023-03-17

**Authors:** Antonio Martínez-Murcia, Adrian Garcia-Sirera, Aaron Navarro, Laura Pérez

**Affiliations:** 1 Department of Microbiology, University Miguel Hernández, 03312 Orihuela, Spain; 2 Genetic PCR Solutions™, 03300 Orihuela, Spain

## Insert Table A2

In the original publication [1], a table of “Reference sequences to each variant/lineages” was not included in the “Data Availability Statement”. Now, we would like to include it as Table A2 in Appendix A. The authors state that the scientific conclusions are unaffected. This correction was approved by the Academic Editor. The original publication has also been updated.

**Table A2:** The sequences included in the reference database.

Reference	Variant	Lineage	Reference	Variant	Lineage
NC_045512.2	Reference genome		EPI_ISL_9009322	Delta	AY.4.2
MT358641.1	Wild Type		EPI_ISL_2921532	Lambda	C.37
MW772455.1	Wild Type		EPI_ISL_2957834	Lambda	C.37
MT470142.1	Wild Type		EPI_ISL_2923197	Lambda	C.37
MT192772.1	Wild Type		EPI_ISL_2930541	Lambda	C.37
EPI_ISL_833226	Alpha	B.1.1.7	EPI_ISL_2932985	Lambda	C.37.1
EPI_ISL_832204	Alpha	B.1.1.7	EPI_ISL_6640917	Omicron	BA.1.17
EPI_ISL_833038	Alpha	B.1.1.7	EPI_ISL_6670244	Omicron	BA.1.17
EPI_ISL_833147	Alpha	B.1.1.7	EPI_ISL_6699736	Omicron	BA.1
EPI_ISL_832169	Alpha	B.1.1.7	EPI_ISL_6699770	Omicron	BA.1
EPI_ISL_833043	Alpha	B.1.1.7	EPI_ISL_6704875	Omicron	BA.1
EPI_ISL_660606	Beta	B.1.351	EPI_ISL_11209238	Omicron	XE
EPI_ISL_745123	Beta	B.1.351	EPI_ISL_11401887	Omicron	XE
EPI_ISL_660608	Beta	B.1.351	EPI_ISL_11490660	Omicron	XE
EPI_ISL_700587	Beta	B.1.351	EPI_ISL_11607013	Omicron	XE
EPI_ISL_712081	Beta	B.1.351	EPI_ISL_11878447	Omicron	XE
EPI_ISL_792680	Gamma	P.1	EPI_ISL_6795834	Omicron	BA.2
EPI_ISL_833170	Gamma	P.1	EPI_ISL_8053581	Omicron	BA.2
EPI_ISL_833169	Gamma	P.1	EPI_ISL_8166509	Omicron	BA.2.10
EPI_ISL_833167	Gamma	P.1	EPI_ISL_8253256	Omicron	BA.2
EPI_ISL_792681	Gamma	P.1	EPI_ISL_13399356	Omicron	BA.5.1
EPI_ISL_1004177	Eta	B.1.525	EPI_ISL_13399365	Omicron	BA.5.1
EPI_ISL_1018082	Eta	B.1.525	EPI_ISL_13399411	Omicron	BA.5.1
EPI_ISL_1020303	Eta	B.1.525	EPI_ISL_13399413	Omicron	BA.5.1
EPI_ISL_1024908	Eta	B.1.525	EPI_ISL_13399833	Omicron	BA.5.1
EPI_ISL_1035823	Eta	B.1.525	EPI_ISL_13402144	Omicron	BA.4
EPI_ISL_2726706	Delta	AY.4	EPI_ISL_13399316	Omicron	BA.4
EPI_ISL_2726793	Delta	AY.75	EPI_ISL_13399339	Omicron	BA.4
EPI_ISL_2727568	Delta	AY.44	EPI_ISL_13402674	Omicron	BA.4.1.1
EPI_ISL_2727864	Delta	AY.46	EPI_ISL_13405368	Omicron	BA.4.1
EPI_ISL_2727605	Delta	AY.75			

## Text Correction

With regard to the insert of Table A2, there are two places that need to be corrected in the text. “Data availability statement section” should be replaced with “Table A2” in Section 2.3. Partial S-Gene Sequencing and Phylogenetic Analysis, paragraph 1, and Section 3.1. In Silico Specificity of the S-Gene 921 BP-Fragment, paragraph 1.
